# Autophagy protein ATG7 is a critical regulator of endothelial cell inflammation and permeability

**DOI:** 10.1038/s41598-020-70126-7

**Published:** 2020-08-13

**Authors:** Mohammad Shadab, Michelle Warren Millar, Spencer A. Slavin, Antony Leonard, Fabeha Fazal, Arshad Rahman

**Affiliations:** Department of Pediatrics, Lung Biology and Disease Program, University of Rochester School of Medicine and Dentistry, 601 Elmwood Avenue, Box 850, Rochester, NY 14642 USA

**Keywords:** Autophagy, Cell signalling

## Abstract

Endothelial cell (EC) inflammation and permeability are critical pathogenic mechanisms in many inflammatory conditions including acute lung injury. In this study, we investigated the role of ATG7, an essential autophagy regulator with no autophagy-unrelated functions, in the mechanism of EC inflammation and permeability. Knockdown of ATG7 using si-RNA significantly attenuated thrombin-induced expression of proinflammatory molecules such as IL-6, MCP-1, ICAM-1 and VCAM-1. Mechanistic study implicated reduced NF-κB activity in the inhibition of EC inflammation in ATG7-silenced cells. Moreover, depletion of ATG7 markedly reduced the binding of RelA/p65 to DNA in the nucleus. Surprisingly, the thrombin-induced degradation of IκBα in the cytosol was not affected in ATG7-depleted cells, suggesting a defect in the translocation of released RelA/p65 to the nucleus in these cells. This is likely due to suppression of thrombin-induced phosphorylation and thereby inactivation of Cofilin1, an actin-depolymerizing protein, in ATG7-depleted cells. Actin stress fiber dynamics are required for thrombin-induced translocation of RelA/p65 to the nucleus, and indeed our results showed that ATG7 silencing inhibited this response via inactivation of Cofilin1. ATG7 silencing also reduced thrombin-mediated EC permeability by inhibiting the disassembly of VE-cadherin at adherens junctions. Together, these data uncover a novel function of ATG7 in mediating EC inflammation and permeability, and provide a mechanistic basis for the linkage between autophagy and EC dysfunction.

## Introduction

Endothelial cell (EC) inflammation and permeability represent two major pathogenic features of many inflammatory conditions including acute lung injury (ALI)^1–3^. ECs form the lining of the blood vessels of many organs such as the lung, heart, brain, kidney, and liver etc. Thus, ECs play an important role as a gate-keeper, preserving vascular integrity and providing a natural barrier to circulating blood, together maintaining homeostasis^4,5^. Previous studies on EC inflammation and barrier function have shown that vascular EC exposed to bacterial, chemical, and mechanical insults secrete inflammatory and chemotactic molecules, and demonstrate loss of barrier integrity^6^. Among the major causes for the exhibition of such inflammation and barrier disruption include activation of the transcription factor NF-κB and disassembly of adherens junctions (AJs)^7,8^. NF-κB is a ubiquitously expressed family of transcription factors which play important roles in various processes including inflammation, cell proliferation, differentiation, and survival^9,10^. The NF-κB family is comprised of five members: Rel (c-Rel), Rel A (p65), Rel B, NF-κB1 (p105/p50), and NF-κB2 (p100/p52)^11,12^. In inactive conditions NF-κB remains in the cytoplasm while bound to its inhibitory protein IκBα^9^. During an inflammatory response, IκBα undergoes phosphorylation at Ser32 and Ser36 leading to its proteasome-mediated degradation and subsequent release of NF-κB (predominantly RelA/p65 homodimer in EC)^13,14^. This allows NF-κB to translocate to the nucleus and transcribe target proinflammatory genes including adhesion molecules, cytokines, and chemokines^12,15,16^. Inhibition of NF-κB signaling has been shown to improve lung function in an endotoxemic model by decreasing lung inflammation and endothelial permeability^17,18^, indicating that the regulation of this signaling is important in the progression of ALI.

EC barrier function is essential for tissue-fluid homeostasis. EC provide a single-layer interface in the capillaries and allow selective passage of fluid, ions, and other molecules between the blood and interstitial space^19–21^. The AJ protein vascular endothelial (VE)-cadherin helps to maintain this selectively-permeable barrier by forming trans-interactions between adjacent ECs. Hence, disruption in endothelial barrier function occurs when the AJs are disassembled at the cell surface. Two main causes for VE-cadherin disassembly are the downregulation of VE-cadherin protein expression that leads to its loss at the cell surface, or tension induced by actin-myosin mediated contractile forces that pull and disassemble VE-cadherin interactions^21–23^. It has been observed that many proinflammatory agents induce actin stress fiber formation in EC, and as such it has been proposed that actin-myosin interactions and subsequent VE-cadherin disassembly are responsible for EC permeability induced by proinflammatory mediators^2,24,25^.

Recent studies have shown an important link between inflammation and autophagy^26,27^. Autophagy is a cellular process that facilitates the recycling of intracellular components like organelles and proteins^28^. It plays a vital role when nutrients are scarce by utilizing the damaged organs or proteins to provide an alternative source of energy^28–31^. During this process, double membrane vesicles called autophagosomes are formed to sequester damaged proteins or organelles. These structures further fuse with lysosomes that contain acidic enzymes to degrade the damaged molecules^28,30,31^. Autophagy is a complicated and highly regulated process which occurs in sequential stages (initiation, nucleation, elongation, and maturation) and involves numerous (> 30) autophagy related proteins including Beclin1 and ATG7^31,32^. Notably, ATG7 is an essential regulator of autophagy with no described autophagy-unrelated functions^31,33,34^. One important step in autophagy is the cleavage of LC3-I to LC3-II through the coordinated activity of several proteins including ATG-5, ATG7, ATG10, and ATG12 and the subsequent incorporation of LC3-II into the autophagosome membrane^31,35^. As cleavage and conjugation of LC3-I to LC3-II occurs only after induction of autophagy, detection of LC3-II has now become a reliable readout for activation of the autophagic process.

Previous studies using proinflammatory agonists have revealed a close relationship between EC inflammation and barrier disruption^36–38^. However, the role of autophagy in these joint processes is poorly understood. We recently established a mechanistic link between autophagy and EC inflammation and permeability by knocking down the autophagy protein Beclin1^39^. However, in addition to an important role of Beclin1 in autophagy, it is also involved in other cellular processes including cell death^40^. Hence, to definitively establish that autophagy is intimately linked to EC dysfunction, we determined the role of ATG7, an essential autophagy regulator with no autophagy-unrelated functions^34^^,^ in EC inflammation and permeability. We found that ATG7 mediates thrombin-induced EC inflammation by regulating RelA/p65 nuclear translocation via cofilin1-dependent alterations in actin cytoskeleton. We also discovered that ATG7 plays a key role in thrombin-induced EC permeability by inducing VE-cadherin disassembly at AJs. Together, these data reveal ATG7 as a novel mechanistic link between autophagy and EC dysfunction (EC inflammation and permeability).

## Results

### ATG7 is required for EC autophagy

**Figure 1:**
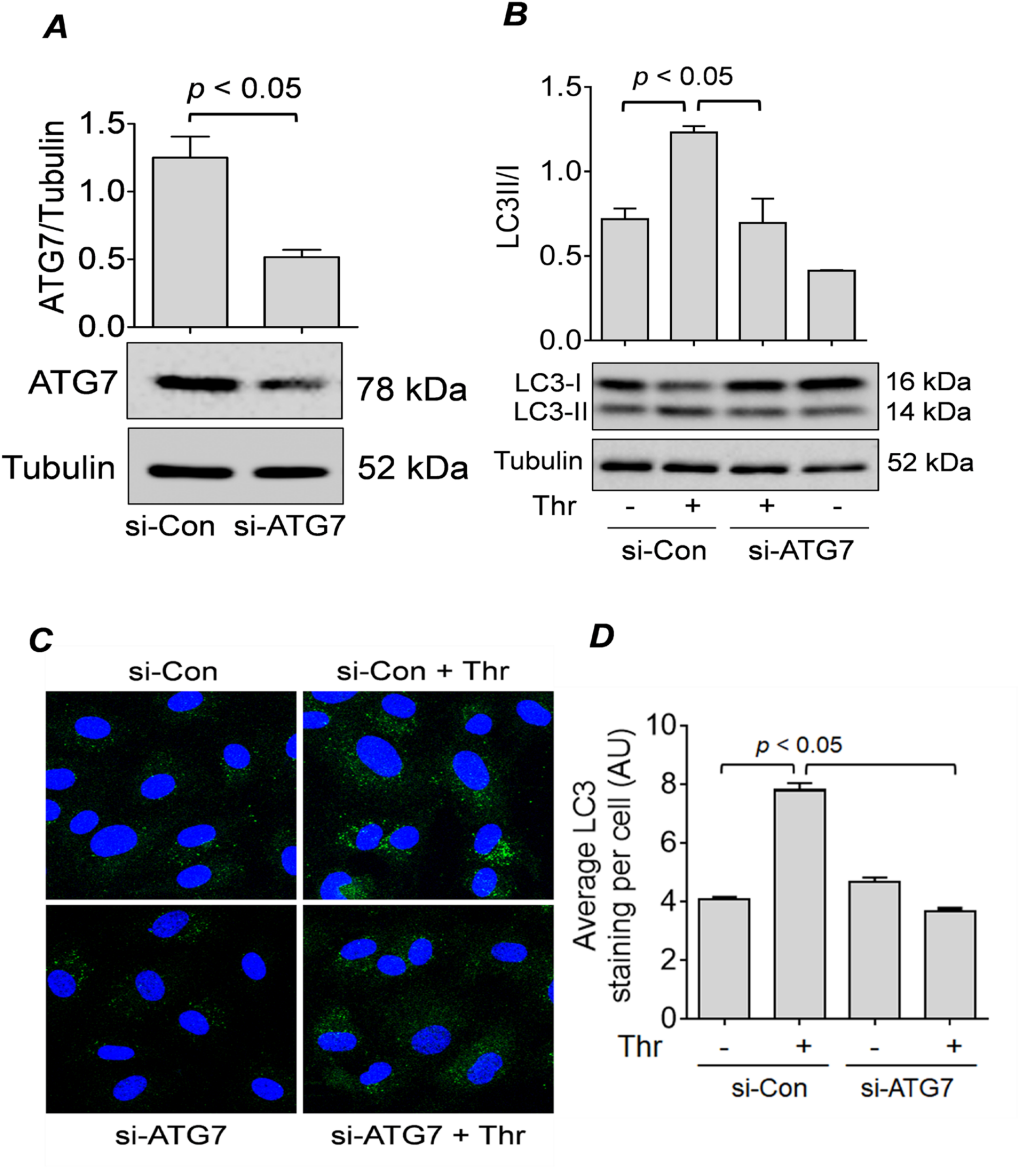
ATG7 knockdown inhibits thrombin-induced autophagy in EC. (**A**) HPAEC were transfected with non-targeting siRNA (si-Con) or siRNA targeting ATG7 (si-ATG7). Cells were lysed after 48 h and analyzed by Western blot for ATG7 levels, and tubulin was used as a loading control. Error bars represent mean ± S.E. (n = 5 for each condition). (**B**) HPAEC transfected with si-Con or si-ATG7 as in (**A**) were treated with thrombin (Thr; 5 U/ml) for 1 h and lysed for Western blot analysis of LC3I/II and tubulin. Error bars represent mean ± S.E. (n = 3 for each condition). (**C**) si-Con and si-ATG7-transfected HPAEC were treated with thrombin (Thr; 5 U/ml) for 1 h and fixed. Cells were stained with anti-LC3 antibody (green) and DAPI (blue) to mark nuclei. Images represent three independent experiments. (**D**) Average LC3 puncta staining per cell represented as mean fluorescence intensity with arbitrary units (AU). Error bars represent mean ± S.E. (n = 3 with 35–45 cells were analyzed per condition).

LC3-II is a well-known marker for autophagosome formation, and lysosomal turnover of LC3-II indicates the rate of autophagic activity^41^. To determine how ATG7 silencing in EC affects thrombin-induced autophagic activity, we monitored the level of LC3-II through Western blot and the formation of LC3 puncta by confocal microscopy. First, we confirmed that there was a significant depletion in the expression of ATG7 in HPAEC by transfection with ATG7 siRNA (si-ATG7) compared to control non-targeting siRNA (si-Con) (Fig. 1A). We also found that thrombin challenge did not affect the levels of ATG7 in cells transfected with si-ATG7 or si-Con (Fig. S1). Next, we showed that upon thrombin challenge, EC exhibited increased conversion of LC3-I to LC3-II, which decreased significantly in ATG7-deficient cells (Fig. 1B). Further, we showed that thrombin treatment of the cells transfected with si-Con led to an increased number of LC3 puncta, and this induction was significantly diminished in cells transfected with si-ATG7 (Fig. 1C,D). Together, these data suggest that knockdown of ATG7 significantly inhibits the thrombin-induced autophagy in EC.

### ATG7 mediates thrombin-induced inflammation via NF-κB activation

**Figure 2:**
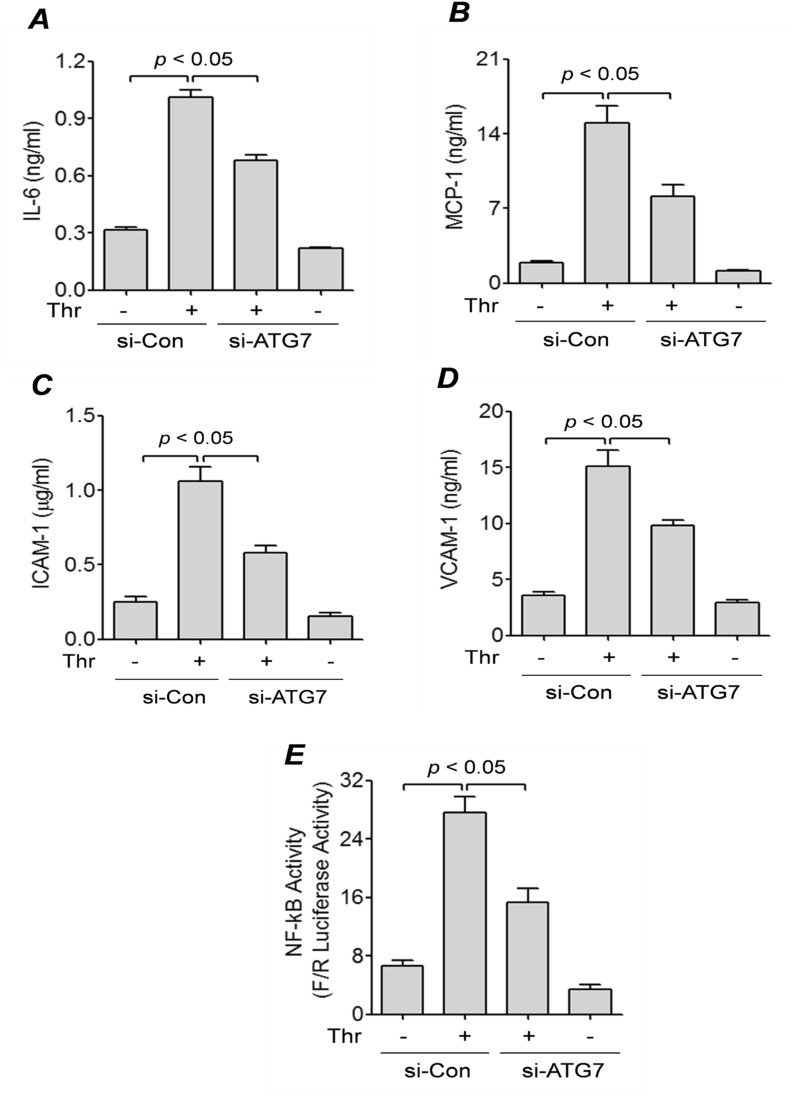
ATG7 knockdown inhibits thrombin-mediated expression of inflammatory proteins and NF-κB activity. HPAEC were transfected with si-Con or si-ATG7 for 48 h and then treated with thrombin (5 U/ml) for 6 h. (**A**,**B**) Conditioned media was collected from the cells and ELISAs were performed for (*A*) IL-6, (*B*) MCP-1. Error bars represent mean ± S.E. (n = 5 for each condition). (**C&D**) Cell lysates were analyzed by ELISA for (*C*) ICAM-1 and (*D*) VCAM-1 levels. Error bars represent mean ± S.E. (n = 5 for each condition). (**E**) HPAEC were transfected with si-Con or si-ATG7 for 24 h, then transfected with NF-κBLUC and *Renilla* LUC constructs as described in the “Materials and Methods”. The cells were then treated with thrombin (5 U/ml) for 6 h and cell extracts were assayed for *Firefly* and *Renilla* luciferase activities. *Renilla* luciferase was used as an internal control for transfection efficiency. Error bars represent mean ± S.E. (n = 6 for each condition).

Inflammation in EC is associated with the production of several proinflammatory mediators including IL-6, MCP-1, ICAM1, and VCAM-1^39,42^. To investigate whether ATG7 has a role in EC inflammation, we treated control or ATG7-depleted cells with thrombin for 6 h and measured the level of secreted IL-6 and MCP-1 using ELISA. We found that compared to control cells, ATG7-depleted cells secreted significantly lower levels of IL-6 and MCP-1 into the culture supernatant (Fig. 2A,B). We also determined if ATG7 knockdown influences the levels of adhesion molecules (ICAM-1 and VCAM-1) in EC. ATG7-depleted cells showed a marked decrease in their ability to produce ICAM-1 and VCAM-1 when challenged with thrombin (Fig. 2C,D). These results reveal ATG7 as an important determinant of EC inflammation.

The transcription factor NF-κB is an important mediator of EC inflammation^7^. Thus, to determine if ATG7 affects EC inflammation via activation of the NF-κB pathway, we measured the level of NF-κB activity in control and ATG7-depleted cells using the dual-Luciferase Reporter Assay system. We found that while thrombin-induced NF-κB activity was increased in control cells, it was significantly diminished in ATG7-depleted cells (Fig. 2E). Together, these findings suggest that ATG7 regulates EC inflammation via NF-κB activation.

### ATG7 is required for thrombin-induced nuclear translocation and subsequent DNA binding of RelA/p65

**Figure 3:**
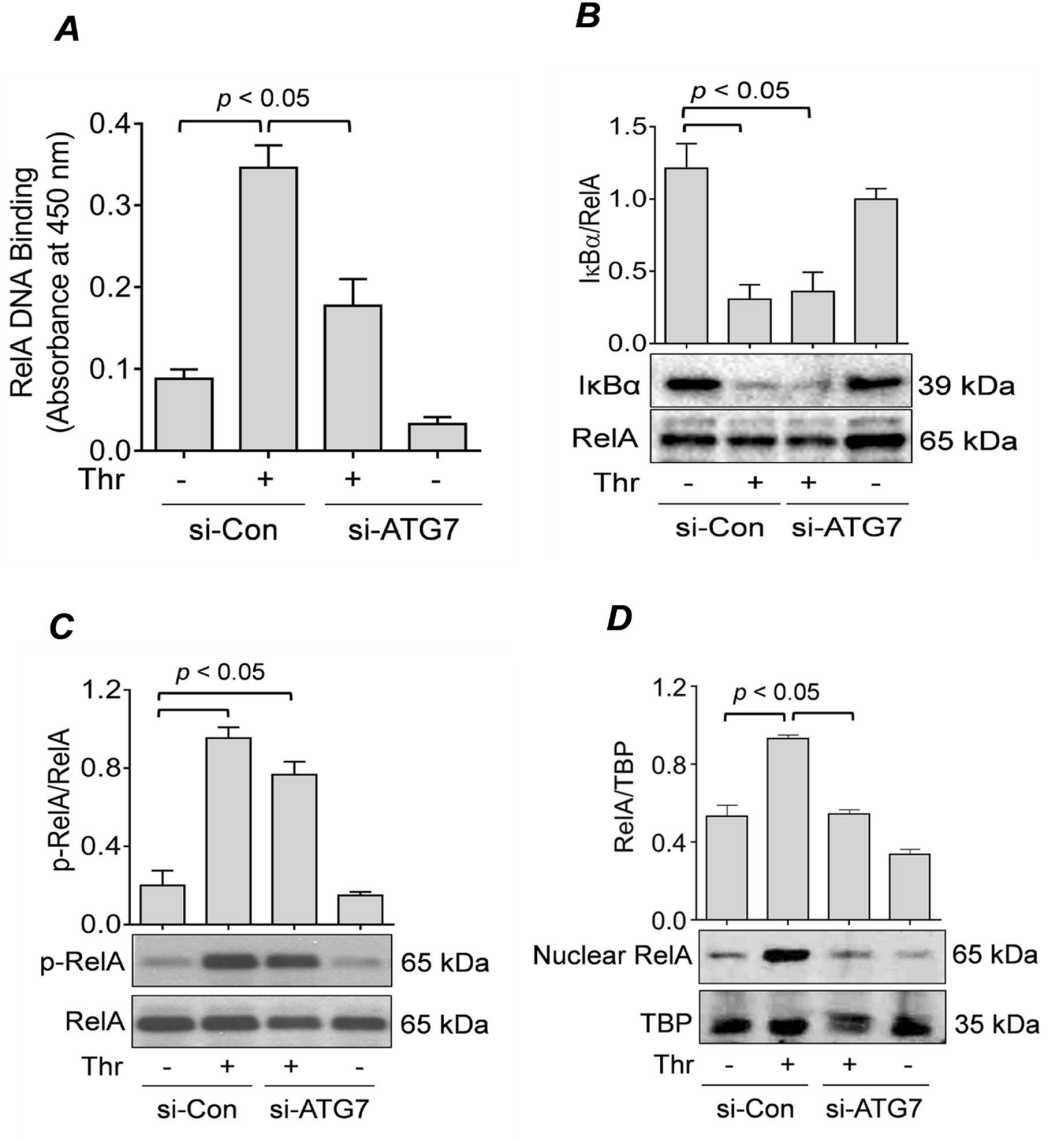
ATG7 knockdown prevents RelA/p65 nuclear translocation and DNA binding, but not IκBα degradation or RelA/p65 phosphorylation. (**A**) HPAEC were transfected for 48 h with si-Con or si-ATG7 and then treated with thrombin (5 U/ml) for 1 h. Nuclear extracts were obtained and processed according to an ELISA-based DNA binding assay kit as described in the “Materials and Methods” section. Error bars represent mean ± S.E. (n = 4 for each condition). (**B&C**) HPAEC were transfected with si-Con or si-ATG7 for 48 h and treated with thrombin (5 U/ml) for 1 h. Total cell lysates were analyzed by Western blot for (*B*) IκBα levels and (*C*) RelA/p65 phosphorylation. RelA/p65 was used as a loading control. Error bars represent mean ± S.E. (n = 3–4 for each condition). (**D**) HPAEC were transfected for 48 h with si-Con or si-ATG7 and treated with thrombin (5 U/ml) for 1 h. Nuclear extracts were obtained and analyzed by Western blot to measure the level of RelA/p65, and TATA-binding protein (TBP) was used as a loading control for nuclear protein. Error bars represent mean ± S.E. (n = 3–4 for each condition).

We have shown previously that upon thrombin challenge of EC, the NF-κB RelA/p65 homodimer translocates to the nucleus and exhibits maximal DNA binding after 1 h of stimulation^15,43,44^. Thus, to assess whether ATG7 knockdown regulates NF-κB activity by affecting DNA binding activity of RelA/p65 in the nucleus, we treated control and ATG7-depleted cells with thrombin for 1 h, and then extracted nuclear extracts to perform a DNA binding assay. We found that there was a significant increase in the DNA binding activity of RelA/p65 in cells transfected with si-Con, which diminished significantly in si-ATG7-transfected cells (Fig. 3A).

Degradation of IκBα and phosphorylation of RelA/p65 are key events leading to nuclear translocation and transcriptional capacity of RelA/p65^44,45^. Hence, we determined whether ATG7 knockdown affects IκBα degradation and RelA/p65 Ser536 phosphorylation upon thrombin treatment. Surprisingly, we found that ATG7 knockdown did not inhibit IκBα degradation (Fig. 3B) or RelA/p65 phosphorylation upon thrombin challenge (Fig. 3C). Together, these results suggest that ATG7 knockdown-mediated inhibition of RelA/p65 nuclear DNA binding and transcriptional activity is independent of IκBα degradation and RelA/p65 phosphorylation.

We have recently shown that silencing of another autophagy protein Beclin1 leads to an impaired translocation of RelA/p65 to the nucleus upon thrombin challenge^39^. This raises possibility that the impaired DNA binding in ATG7 deficient cells may be attributed to disruption of RelA/p65 nuclear translocation. Thus, to investigate whether ATG7 knockdown also abrogates RelA/p65 translocation, we treated cells transfected with si-Con or si-ATG7 with thrombin for 1 h^44^ and then extracted nuclear lysates to perform immunoblotting. We found that there was a significant decrease in thrombin-induced RelA/p65 nuclear translocation in ATG7-deficient cells (Fig. 3D). These results suggest that ATG7 knockdown inhibits RelA/p65 DNA binding by hindering its translocation to the nucleus, thereby uncovering a novel mechanism for ATG7 regulation of the NF-κB pathway.

### ATG7 mediates thrombin-induced RelA/p65 translocation via Cofilin1 phosphorylation and actin stress fiber formation

**Figure 4:**
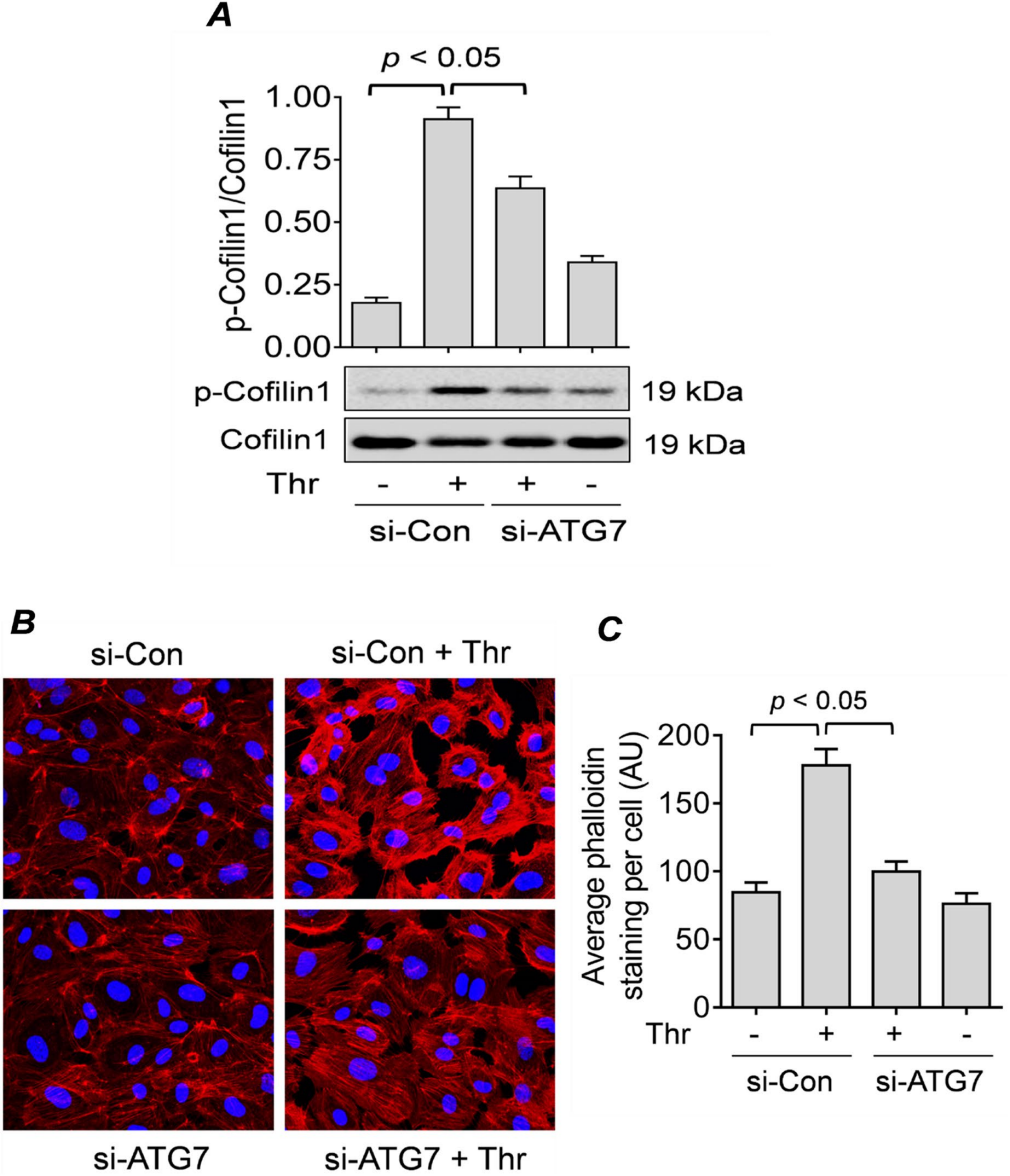
ATG7 knockdown impairs RelA/p65 nuclear translocation by inhibiting Cofilin1 phosphorylation and actin stress fiber formation. (**A**) HPAEC were transfected for 48 h with si-Con or si-ATG7 and treated with thrombin (5 U/ml) for 15 min. Total cell lysates were obtained to measure the level of cofilin1 phosphorylation at Ser3 through Western blot. Total Cofilin1 level was used as a loading control. Error bars represent mean ± S.E. (n = 3–4 for each condition). (**B**) HPAEC were transfected for 48 h with si-Con or si-ATG7 and treated with thrombin (5 U/ml) for 15 min. Actin filaments and nuclei were visualized by staining with Alexa Fluor 594-labeled phalloidin (red) and DAPI (blue), respectively. Images are representative of three experiments. (**C**) Average phalloidin staining per cell represented as mean fluorescence intensity with arbitrary units (AU). Error bars represent mean ± S.E. (n = 3, a total of 30–32 cells were analyzed for each condition).

Previous studies from our lab have shown that thrombin-induced nuclear translocation of RelA/65 requires the phosphorylation and inactivation of Cofilin1 and the generation of actin stress fibers^39,46^. Thus, to determine if this mechanism is involved in the effect of ATG7 silencing on RelA/p65 nuclear translocation, we analyzed Cofilin1 phosphorylation and generation of actin stress fiber in cells transfected with si-Con or si-ATG7. We found that phosphorylation/inactivation of cofilin1 (Fig. 4A) and stress fiber formation (Fig. 4B,C) was significantly inhibited in thrombin treated ATG7-depleted cells compared to control cells. These results suggest that the inhibition of thrombin-induced RelA/p65 nuclear translocation by ATG7 knockdown occurs via modulation of Cofilin-1 phosphorylation and generation of actin stress fibers.

### ATG7 knockdown reduces thrombin-induced EC permeability by inhibiting disassembly of VE-cadherin at AJs

**Figure 5:**
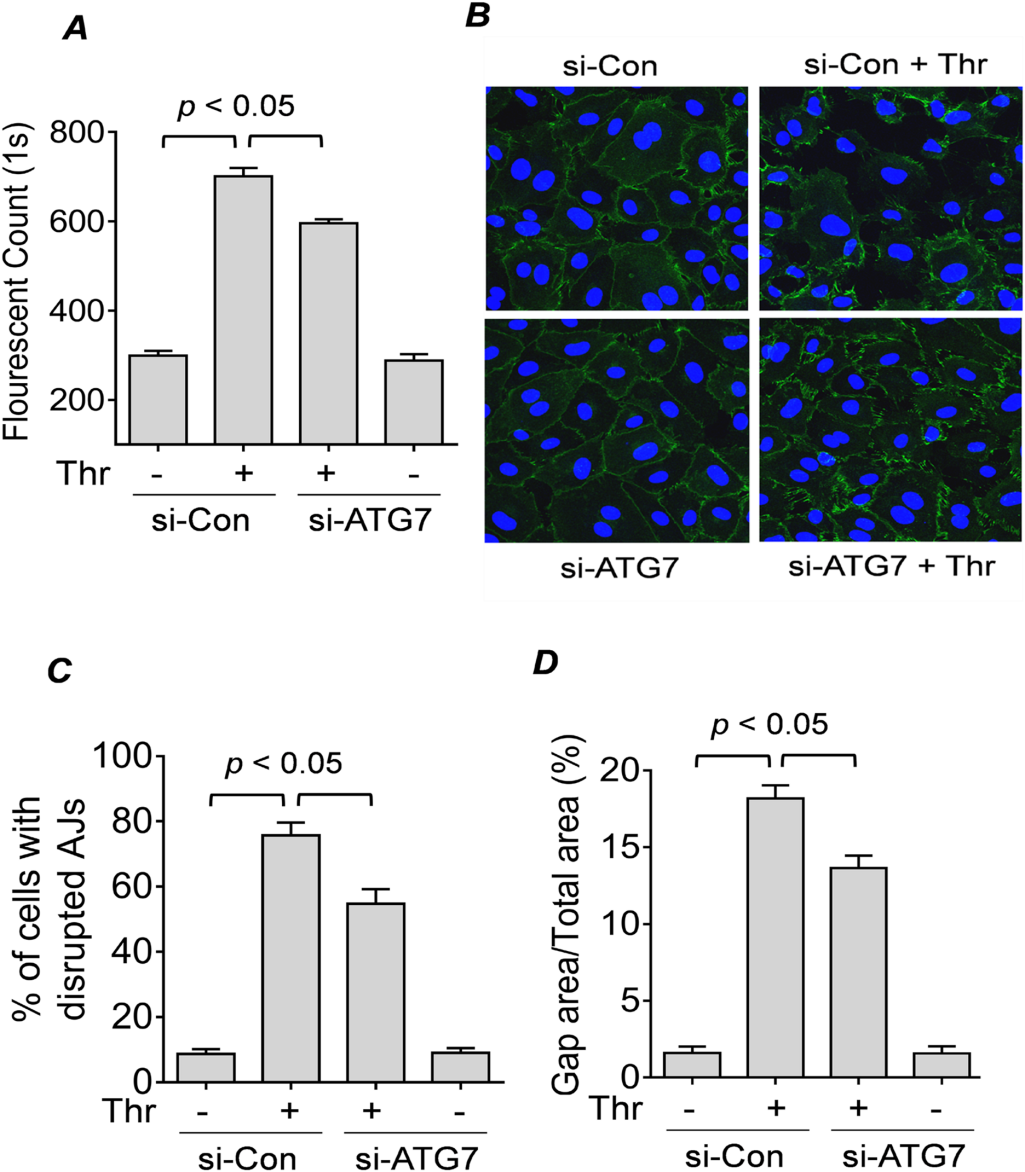
ATG7 knockdown protects against thrombin-induced EC permeability by inhibiting disassembly of VE-Cadherin at AJs. (**A**) HPAEC were transfected with si-Con or si-ATG7 for 24 h. Cells were then seeded on inserts placed in a 24-well plate and allowed to grow to confluence. The cells were treated with thrombin (2.5 U/ml) for 15 min and FITC-Dextran was added directly to the culture media. FITC-Dextran which had passed through the monolayer was measured using a fluorescent plate reader. Error bars represent mean ± S.E. (n = 5 for each condition). (**B**) HPAEC were transfected for 48 h with si-Con or si-ATG7. Cells were treated with thrombin (5 U/ml) for 15 min and non-permeabilized cells were stained with anti-VE-cadherin (green) to mark cell surface VE-cadherin and DAPI (blue) to mark nuclei. Images are representative of three experiments. (**C**) The percent of cells with disrupted adherens junctions (AJs) in (*B*) was counted as described in the “Materials and Methods” section. Error bars represent mean ± S.E. (n = 3 with 40–50 cells were counted in every fields for each conditions). (**D**) The percent gap area in (*B*) was calculated as described in the “Materials and Methods”. Error bars represent mean ± S.E. (n = 3 fields analyzed per condition).

The effect of ATG7 depletion on thrombin-induced actin stress fiber formation (Fig. 4B,C) points to a role of ATG7 in endothelial permeability. To determine the effects of ATG7 knockdown on EC barrier function, we evaluated the permeability of si-Con or si-ATG7 EC monolayers using the FITC-Dextran permeability transwell assay^47^. Results showed that there was a significant decrease in the permeability of the ATG7-depleted cells after thrombin treatment compared to control cells. Moreover, ATG7 silencing alone did not significantly alter the permeability in untreated cells (Fig. 5A). These results suggest that ATG7 knockdown protects against EC permeability caused by thrombin.

The assembly of AJ proteins like VE-cadherin at the cell surface is important for maintaining EC barrier integrity^48^. To determine whether knockdown of ATG7 affects the disassembly of VE-cadherin at AJs after thrombin challenge, we stained EC monolayers for cell surface VE-cadherin and measured interendothelial gap formation in cells transfected with si-Con or si-ATG7. We found that compared to control cells, ATG7 knockdown reduced the disassembly of AJs after thrombin challenge as revealed by retained cell surface VE-cadherin and significantly fewer gaps between the ATG7-deficient cells (Fig. 5B–D). These results show that ATG7 is a critical mediator of thrombin-induced EC permeability by its ability to cause disassembly of VE-cadherin at AJs.

## Discussion

Autophagy is an intracellular recycling process that removes damaged organelles or proteins^30,49^, and provides an alternative source of energy during periods of cellular stress^50^. During this process, the damaged cargo is first entrapped in a double membrane structure known as autophagosome^51^. The autophagosome then fuses with a lysosome to form an autolysosome. As a result, the engulfed cargo is degraded by enzymes provided by the acidic environment of the lysosome^50^. Autophagosome formation occurs in several phases with the coordinated action of several autophagy-related (ATG) proteins. The initiation phase is regulated by several ATG proteins including Beclin1, and the elongation phase is orchestrated by other ATG proteins including ATG7^10,28^. Previous studies have shown that autophagy modulates several cellular processes including cellular survival, inflammation, and immunity^10,28,52^ and is linked to many disease states^26,52–54^. We have recently reported a novel role of autophagy in EC inflammation and barrier disruption through silencing of Beclin1 and use of autophagy inhibitor 3-methyladenine (3-MA) in a mouse model of ALI^39,55^. Although Beclin1 is an important upstream regulator of autophagy, it is also involved in other cellular processes including cell death^40^. Unlike Beclin1, ATG7 is an essential autophagy protein with no described autophagy-unrelated functions^33,34^. Hence, to definitively establish the linkage between autophagy and EC dysfunction, in this study we evaluated whether ATG7 knockdown plays a role in the mechanism of EC inflammation and barrier disruption, a more specific method of autophagy inhibition than either Beclin1 knockdown or 3-MA treatment. It is also important to note that unlike Beclin1 knockdown or 3-MA treatment which target the initiation phase of autophagy, ATG7 knockdown targets the elongation phase. We demonstrated that ATG7 is indispensable for EC inflammation upon thrombin challenge because knockdown of ATG7 leads to attenuation of this response. We next showed the mechanistic basis of this reduced EC inflammation, where NF-κB activity was inhibited as a result of impaired translocation of RelA/p65 from the cytosol to the nucleus despite unaffected levels of IκBα degradation and RelA/p65 Ser536 phosphorylation. We also showed that knocking down ATG7 protects EC permeability after thrombin treatment by inhibiting the disassembly of VE-cadherin at AJs and formation of actin stress fibers. Altogether, our findings reveal an important role of ATG7 in modulating EC function.

The lysosomal turnover of the autophagosomal marker LC3-II has become a reliable method for monitoring autophagy^56^. Our results demonstrate a significant reduction in thrombin-induced formation of LC3-II in ATG7-depleted cells compared to control cells, indicating that ATG7 is an important regulator of thrombin-induced autophagy in EC. Studies have shown that autophagy has a context-dependent effect on cellular inflammation, and can induce or suppress inflammatory responses depending on the cell type or disease state^57^. However, the relationship between autophagy and inflammation in EC remains poorly understood. To determine how ATG7 expression, and thus autophagy in general, regulates EC inflammation, we determined the levels of the well-known inflammatory molecules including ICAM-1, VCAM-1, IL-6, and MCP-1. Our results showed that ATG7 positively regulates the expression of these inflammatory molecules in response to thrombin, as the knockdown of ATG7 significantly reduced their levels in EC. Interestingly, ATG7 knockdown was also effective in inhibiting IL-6 production in response to lipopolysaccharides (LPS) challenge (Fig. S2). These data show that the proinflammatory role of ATG7 is not restricted to thrombin, and support the idea that it is a general mediator of EC inflammation. Our previous findings that knockdown of Beclin1 also attenuate EC inflammation upon thrombin challenge^39^ points to autophagy as a common pathway through which Beclin1 and ATG7 regulate EC inflammation.

We have established previously that the NF-κB pathway regulates EC inflammation upon thrombin challenge^7,58^. In the current study, we showed that ATG7 is required for EC inflammation, so we next tested if ATG7 affects EC inflammation by regulating the NF-κB pathway. Using the NF-κB luciferase assay, we demonstrate that the activity of NF-κB is significantly inhibited in ATG7-depleted EC compared to control cells. We next investigated specific steps of the NF-κB signaling pathway and their involvement in ATG7-mediated regulation of NF-κB activity. Our study showed that thrombin-mediated degradation of IκBα and phosphorylation of RelA/p65 were unaffected by ATG7 knockdown. Despite this, ATG7 knockdown inhibited thrombin-induced nuclear activation of RelA/p65, as assessed by DNA binding activity of RelA/p65 in the nucleus. A similar observation was made upon Beclin1 silencing in our previous work^39^. The normal liberation from IκBα and activating phosphorylation, but diminished DNA binding activity of RelA/p65 points to a possible defect in the translocation of the released RelA/p65 to the nucleus. Using Western blot analysis of nuclear EC fractions, we further showed that translocation of RelA/p65 to the nucleus was hindered in ATG7-depleted cells. These observations were similar to what we found with Beclin1 silencing^39^^,^ suggesting that both the proteins affect thrombin-induced nuclear translocation of RelA/p65 in EC. However, unlike Beclin1, ATG7 does not appear to play a role in regulating RelA/p65 phosphorylation, which may be due to autophagy-unrelated effect of Beclin1 or the different stages of autophagy regulated by Beclin1 and ATG7.

The role of actin cytoskeleton in the translocation RelA/p65 from cytosol to nucleus has been previously demonstrated by our group^43^. We have shown that Cofilin-1 phosphorylation/inactivation and the subsequent generation of actin stress fibers are crucial steps that allow RelA/p65 to translocate to the nucleus after thrombin treatment^37,43,46^. Moreover, we have recently shown that knockdown of Beclin1 attenuates thrombin-induced Cofilin1 phosphorylation/inactivation and actin stress fiber formation as a mechanism of inhibiting the nuclear translocation of RelA/p65 in EC^43^. Therefore, using Western blot analysis and immunofluorescence microscopy, we show here that Cofilin1 phosphorylation/inactivation and generation of actin stress fibers were inhibited significantly in ATG7-depleted cells compared to control cells, indicating that both ATG7 and Beclin1 exhibit the same mechanism to prevent RelA/p65 nuclear translocation. These data thus reveal that thrombin-mediated EC inflammation requires autophagy for efficient RelA/p65 nuclear translocation via Cofilin1-dependent generation of actin stress fibers.

The regulation of Cofilin1 phosphorylation and actin stress fiber formation by ATG7 raises the possibility of a crosstalk between ATG7/autophagy and RhoA/ROCK/LIMK1/Cofilin1/Actin pathway. It has been reported that TLR4-induced ATG7/autophagy inhibits enterocyte migration via induction of RhoA-mediated actin stress fibers^59^. Another study by Mondaca-Ruff et al.^60^ showed that angiotensin II induces autophagy via the RhoA/ROCK pathway to cause vascular smooth muscle cells (VSMC) hypertrophy. Thus, ATG7/autophagy can act both upstream and downstream of RhoA/ROCK pathway. Future studies into ATG7-RhoGTPase interactions could reveal new links between autophagy and several important cellular functions including cytoskeletal dynamics, cell adhesion, and EC migration, in addition to EC inflammation.

The ability of ATG7 to regulate actin stress fiber formation also implicates it in EC permeability. Indeed, ATG7 knockdown was effective in inhibiting thrombin-induced EC permeability. Consistent with this, thrombin-induced disassembly of VE-cadherin at AJs, a critical determinant of EC barrier disruption, was also protected in ATG7-depeleted cells. Specifically, we found that ATG7 knockdown inhibited the loss of VE-cadherin at the cell surface and minimized inter-endothelial gap formation after thrombin challenge.

In summary, this study describes a novel function of ATG7 wherein it is required for thrombin-induced EC inflammation. ATG7 silencing significantly reduced thrombin-mediated actin stress fiber generation which is required for the translocation of RelA/p65 to the nucleus to transcribe target inflammatory genes. This study also highlights the importance of ATG7 in EC barrier function. ATG7 knockdown protected EC permeability by inhibiting the disassembly of VE-cadherin at the cell surface in addition to reducing actin stress fiber formation after thrombin treatment. The findings reveal the involvement of ATG7 in EC inflammation and permeability, and establish a role for autophagy in EC dysfunction.

## Materials and methods

### Reagents

Human α-thrombin was purchased from Enzyme Research Laboratories (South Bend, IN). Anti-ATG7, anti-phospho-(Ser536)-RelA/p65 (3033S), anti-phospho-(Ser3)-Cofilin1 antibody (3311S), and anti-Cofilin1 (clone D3F9, 5175S) antibodies were purchased from Cell Signaling Technology (Beverly, MA). Antibodies to RelA/p65 (SC-8008), IκBα (SC-371), and β-actin (SC-47778) were from Santa Cruz Biotechnology (Santa Cruz, CA). An anti-TBP antibody was purchased from Abcam (AB33168, Cambridge, MA). Plasmid maxi kit was from QIAGEN Inc. (Valencia, CA) and diethylaminoethyl (DEAE)-dextran was obtained from Sigma-Aldrich Chemical Company (St. Louis, MO). All other materials were obtained from Thermo Fisher Scientific (Waltham, MA).

### Cell culture

Human pulmonary artery endothelial cells (HPAEC) were purchased from Lonza (Walkersville, MD). They were cultured in 0.1% gelatin-coated flasks as described previously^39^ in endothelial basal medium 2 (EBM2) containing 10% FBS and bullet kit additives (BioWhittaker, Walkersville, MD). Experiments were performed with HPAEC which were between passages 4 and 7.

### siRNA-mediated knockdown of ATG7

Predesigned SMART pool short-interfering RNA duplexes specific for human ATG7 (si-ATG7) and a non-targeting siRNA control (si-Con) from Dharmacon (Lafayette, CO) were used to knockdown ATG7. HPAEC were transfected with si-Con or si-ATG7 using DharmaFect1 siRNA Transfection Reagent (Dharmacon) as described previously^46^. Briefly, HPAEC were grown to 60–70% confluency and transfected with 100 nM siRNA mixed with DharmaFect1 reagent. After 36-48 h, cells were used for experiments.

### NF-κB transcriptional activity

The NF-κB transcriptional activity was measured as described previously^13^. The plasmid pNF-κBLUC containing 5 copies of consensus NF-κB sequences linked to a minimal E1B promoter-*Firefly* luciferase gene was purchased from Stratagene (La Jolla, CA). Briefly, HPAEC were transfected for 24 h with si-Con or si-ATG7 as described above. These cells were re-transfected for 24 h with 5 μg pNF-κB-LUC using 50 µg/ml DAE-dextran in serum-free EBM2 medium as described previously^37^.The transfection mixture also included 0.125 µg pTKRLUC plasmid (Promega Corp., Madison, WI) containing *Renilla* luciferase gene driven by the constitutively active thymidine kinase (TK) promoter, which was used to normalize the transfection efficiency. After 1 h, cells were incubated with 10% dimethyl sulfoxide (DMSO) in serum-free medium for 4 min. The cells were then washed two times with PBS and allowed to grow to confluency in EBM2 medium containing 10% FBS. After treatment with thrombin, cell extracts were prepared and assayed for Firefly and *Renilla* luciferase activities using Promega Biotech Dual Luciferase Assay System (Promega Biotech, Madison, WI). The data were expressed as a ratio of *Firelfly* to *Renilla* luciferase activity.

### Immunoblot analysis

After appropriate treatments, cells were directly lysed in radioimmune precipitation (RIPA) buffer (50 mM Tris–HCl [pH 7.4], 150 mM NaCl, 0.25 mM EDTA [pH 8.0], 5 mM NaF, 1% Triton-X, 1% deoxycholic acid, 1 mM sodium orthovanadate, and protease inhibitor cocktail [Sigma]) or phosphorylation lysis buffer (50 mM HEPES, 150 mM NaCl, 10 mM sodium pyrophosphate, 1 mM EDTA, 200 μM sodium orthovanadate, 100 mM sodium fluoride, 1.5 mM magnesium chloride, 0.5 to 1% Triton X-100, 10% glycerol, 1 mM phenylmethylsulfonyl fluoride [PMSF] supplemented with protease inhibitor cocktail (Sigma) as described^39^. Using SDS-PAGE, cell lysates were resolved and then transferred onto nitrocellulose membranes (BioRad, Hercules, CA, USA). The membranes were blocked with 5% (w/v) nonfat dry milk in Tris-buffered saline (TBS) containing 0.5% Tween-20 for 1 h at room temperature and then probed with primary antibody overnight at 4 °C at a dilution recommended by the manufacturer. After washing for three times in TBST, the membranes were incubated with horseradish peroxidase (HRP)-conjugated secondary antibody and detected by enhanced chemiluminiscence (ECL) detection system according to the manufacturer’s instructions. Representative blots presented in the results section come from the same membrane which may have more samples in various groups.

### Immunofluorescence

HPAEC grown to confluence on gelatin-coated coverslips were subjected to immunofluorescence staining as described previously^43^. VE-cadherin antibody (BD Biosciences, San Jose, CA), LC3 antibody (Cell signaling Technology, Danvers, MA, USA), Alexa Fluor 594-phalloidin (Invitrogen, Grand Island, NY, USA) and DAPI were used to visualize AJs, LC3 puncta, F-actin filaments and nuclei, respectively. Following staining the coverslips were rinsed in PBS and mounted on slides using Vectashield mounting media (Vector Laboratories, Lincolnshire, IL). Images were captured using Axio Imager M2m confocal microscope (Zeiss). ImageJ tool was used to measure LC3 and phalloidin staining intensity of the individual cells and the average fluorescence from 30–45 cells in a field was obtained. The average fluorescence value per cell was presented as bar graphs. The percentage of cells with disrupted adherens junctions (AJs) was calculated by counting cells with discontinuous cell surface VE-cadherin staining and normalized to the total number of cells (40–50) in the field. The percentage of cells with a disrupted VE-cadherin staining border was averaged and presented as bar graphs. To measure gap formation between cells, gaps or areas without cell coverage were outlined and measured using ImageJ tool and then the area of gaps in each field was normalized to the size of the field. The percentage of gap area was averaged and presented as bar graphs.

### ELISA

The levels of IL-6, MCP-1, ICAM-1 and VCAM-1 in HPAEC were determined using ELISA kits from R&D Systems (Minneapolis, MN) according to manufacturers’ recommendations.

### Assessment of RelA/p65 DNA binding activity in nuclear extract

After appropriate treatments, cells were washed twice with ice-cold Tris-buffered saline and processed further as per the protocol described earlier^39^. The cells were then dislodged using cell scraper while remaining in 1 ml Tris-buffered saline and subjected to centrifugation. The cell pellet thus collected was resuspended in 400 µl of buffer A containing 10 mM HEPES [pH 7.9], 10 mM KCl, 0.1 mM EDTA, 0.1 mM EGTA, 1 mM [DTT], and 0.5 mM PMSF. After 15 min, NP-40 was added to achieve a final concentration of 0.6%, and the samples were centrifuged and supernatants containing the cytoplasmic proteins were collected. The nuclear pellet was incubated in 50 µl of buffer B (20 mM HEPES [pH 7.9], 0.4 M NaCl, 1 mM DTT, 1 mM EDTA, 1 mM EGTA, and 1 mM PMSF) at 4 °C for 30 min. The lysates were then centrifuged to collect the supernatants containing the nuclear proteins. Equal amount of nuclear proteins was analyzed for RelA/p65 nuclear translocation by immunoblotting. The DNA binding activity of RelA/p65 was determined using an ELISA-based DNA binding assay kit (Cayman Chemical, Ann Arbor, MI) as described^39^.

### Measurement of endothelial permeability

Endothelial permeability to macromolecules was assessed using an In Vitro Vascular Permeability Assay (FITC-Dextran) kit (ECM644: Millipore) following the manufacturers protocol. Briefly, HPAEC were transfected with si-Con or si-ATG7. After 48 h, 20,000 cells were transferred to each transwell insert, and allowed to grow to confluence. Cells were then treated with thrombin for 20 min and then FITC-Dextran was added directly to the culture media. Media from the receiver tray was collected and fluorescent intensities were measured on a fluorescent plate reader using an excitation wavelength of 485 nm and emission wavelength of 535 nm.

### Statistical analysis

All data comparisons were tested for significance using one-way ANOVA, followed by Tukey post-test for multiple groups and a Student's *t-*test for two groups. GraphPad Prism 6 (GraphPad Software, San Diego) was used for all statistical analyses and data presented as mean ± SE. A *p* value < 0.05 was considered statistically significant.

## Supplementary information

Supplementary information.
