# Supplementary material for: Autophagy protein ATG7 is a critical regulator of endothelial cell inflammation and permeability
Source: Sci Rep. 2020 Aug 13;10:13708. doi: 10.1038/s41598-020-70126-7 (PMC7426828; doi:10.1038/s41598-020-70126-7)
Supplement: Supplementary file 1 — Supplementary information. [file 41598_2020_70126_MOESM1_ESM.pdf]

Supplemental material for manuscript

AUTOPHAGY PROTEIN ATG7 IS A CRITICAL REGULATOR OF ENDOTHELIAL CELL INFLAMMATION AND PERMEABILITY

Mohammad Shadab, Michelle Warren Millar, Spencer A. Slavin, Antony Leonard, Fabeha Fazal, Arshad Rahman*

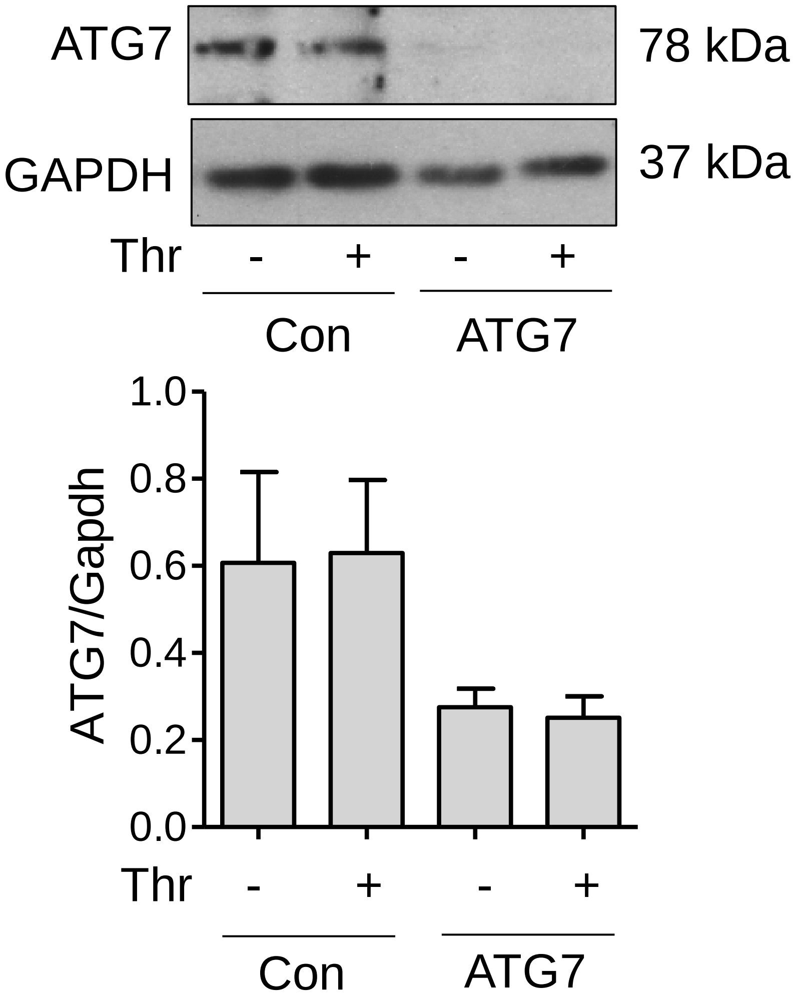

Figure S1. Effect of thrombin on the expression of ATG7 in EC. HPAEC were transfected with non-targeting siRNA (si-Con) or siRNA targeting ATG7 (si-ATG7). After 48 h of transfection cells were treated with thrombin (5 U/ml) and lysed. The samples were analyzed by Western blot for ATG7 levels, and GAPDH was used as a loading control. Error bars represent mean ± S.E. (n=3 for each condition).

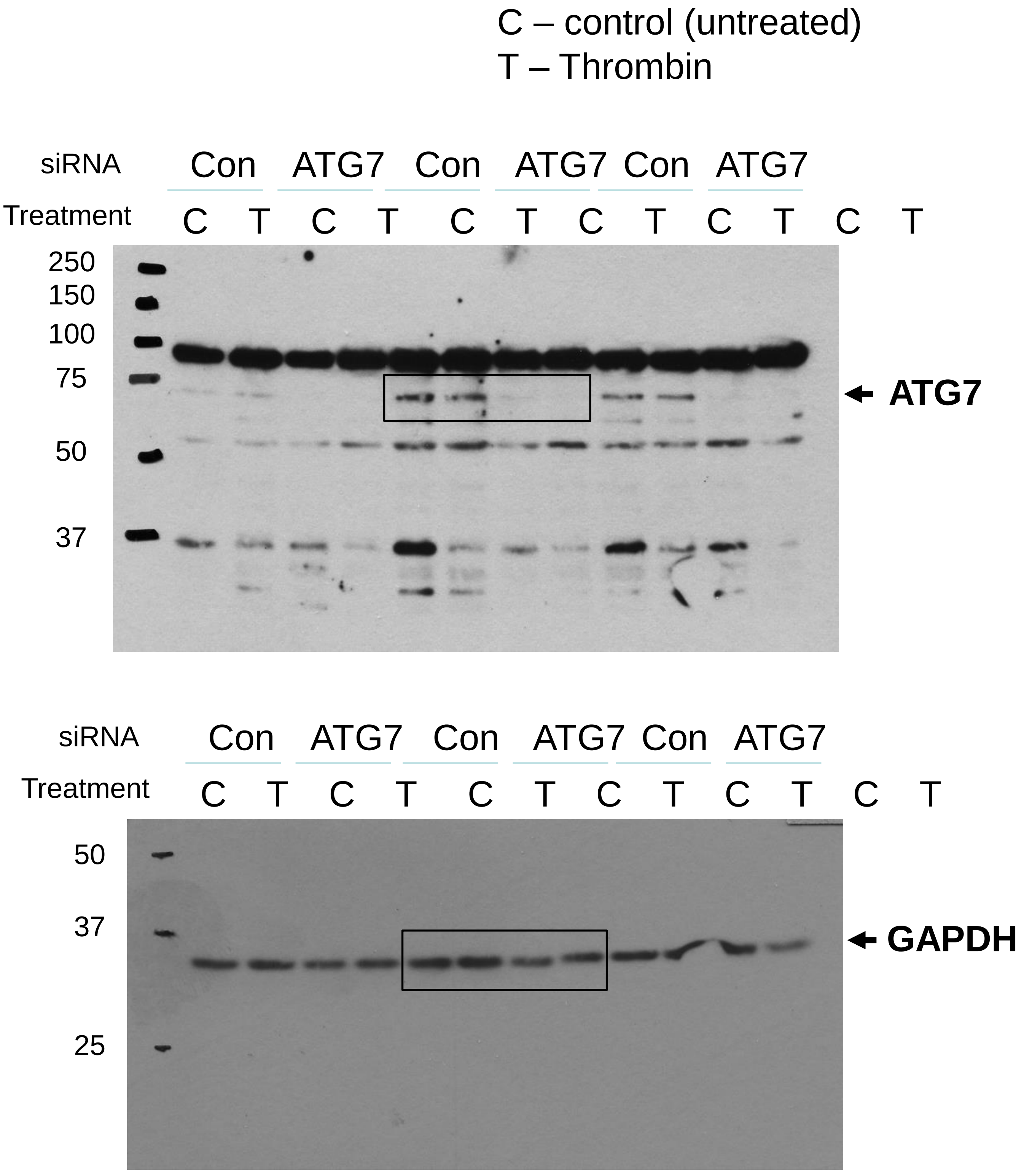

Uncropped Figure S1. Uncropped versions of blots shown in Figure S1. The area under the box was used in Fig. S1.

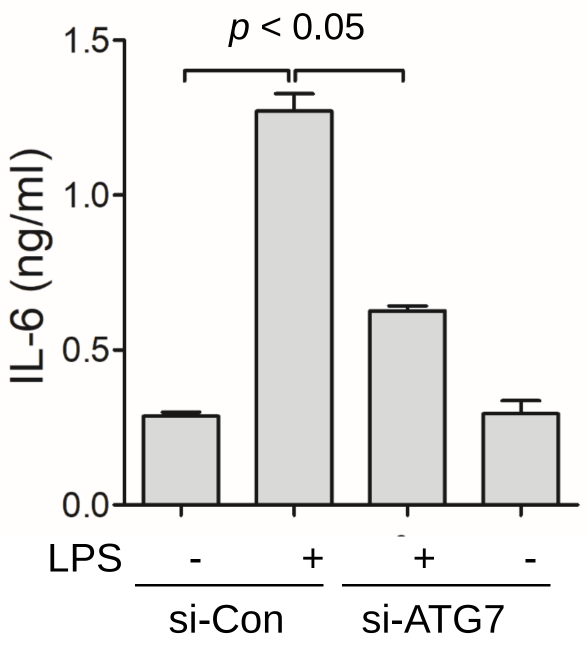

Figure S2. ATG7 knockdown inhibits LPS-induced increase in IL-6 level. HPAEC were transfected with si-Con or si-ATG7 for 48 h and then treated with LPS (1 µg/ml) for 6 h. Conditioned media was collected from the cells and ELISA was performed for IL-6. Error bars represent mean ± S.E. (n=5 for each condition).

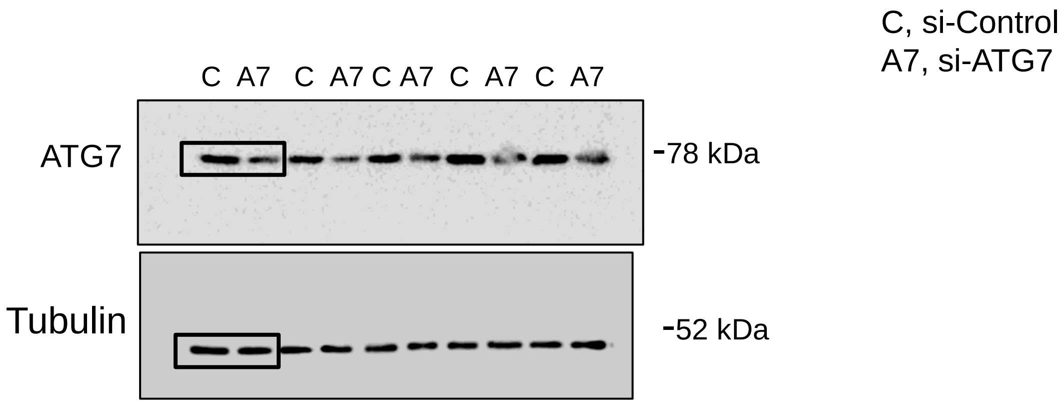

Figure S3A. Uncropped versions of blots shown in Figure 1A.
The area under the box was used in Fig. 1A

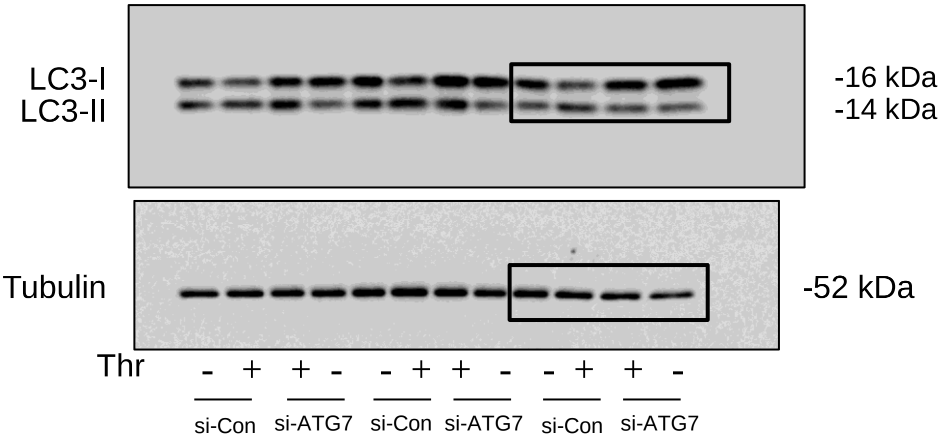

Figure S3B. Uncropped versions of blots shown in Figure 1B. The area under the box was used in Fig. 1B.

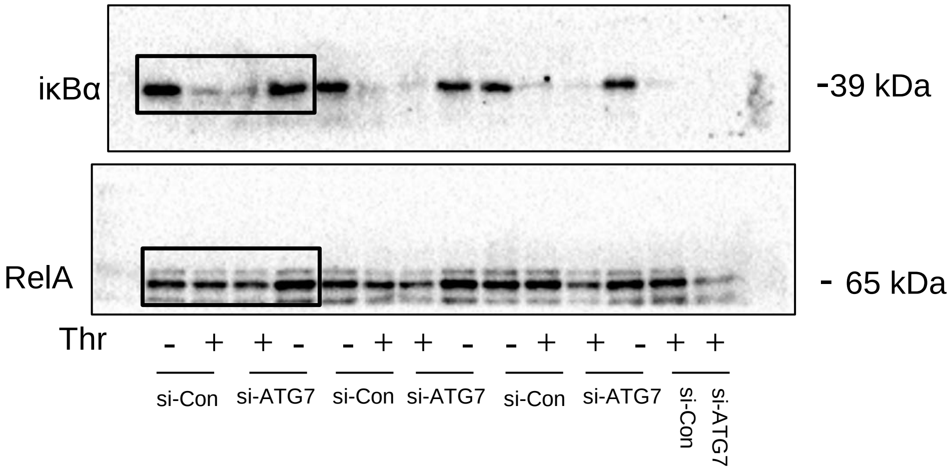

Figure S4A. Uncropped versions of blots shown in Figure 3B. The area under the box was used in Fig. 3B.

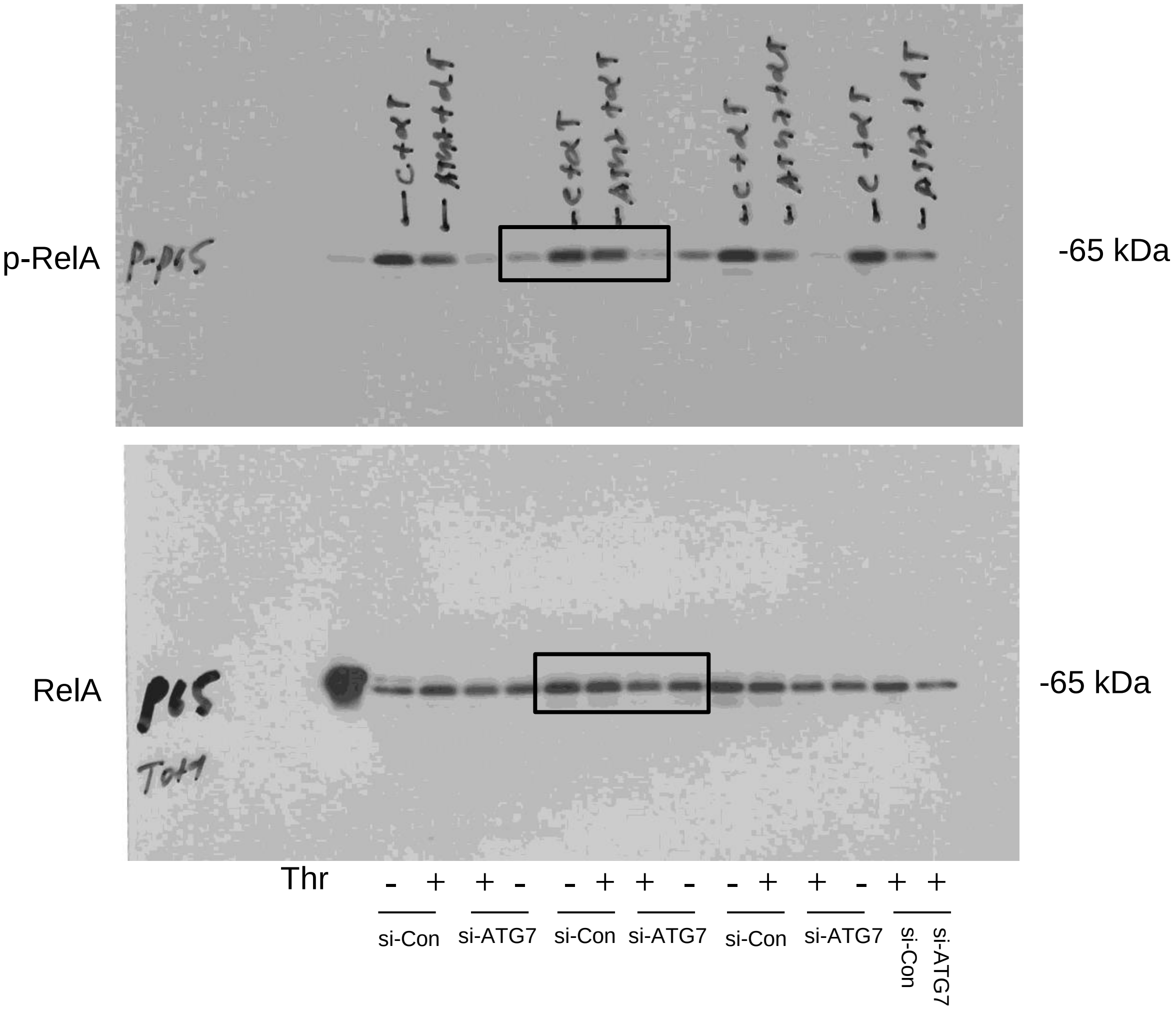

Figure S4B. Uncropped versions of blots shown in Figure 3C. The area under the box was used in Fig. 3C.

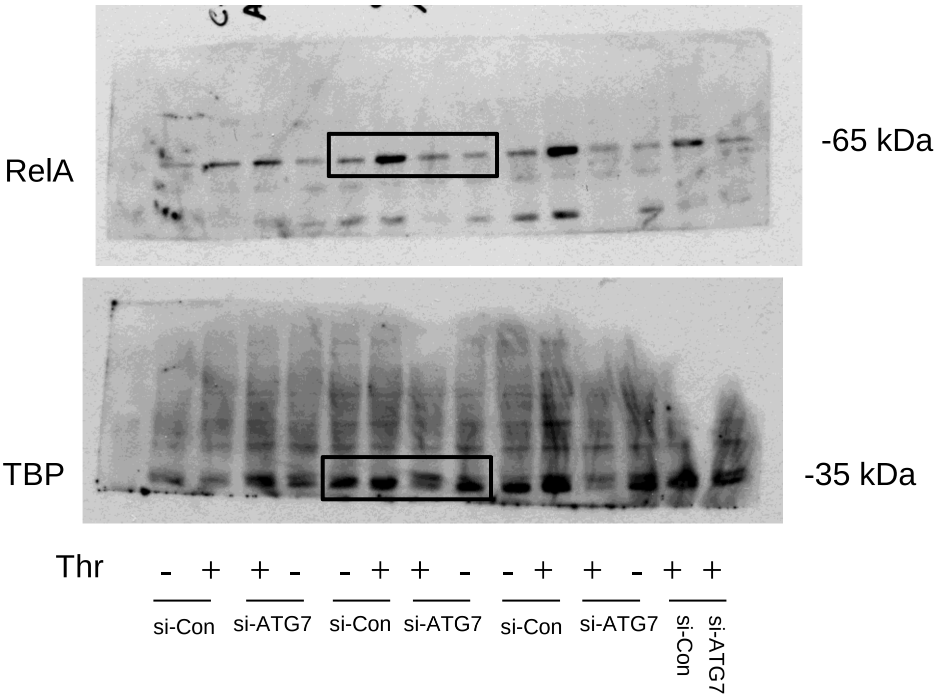

Figure S4C. Uncropped versions of blots shown in Figure 3D. The area under the box was used in Fig. 3D.

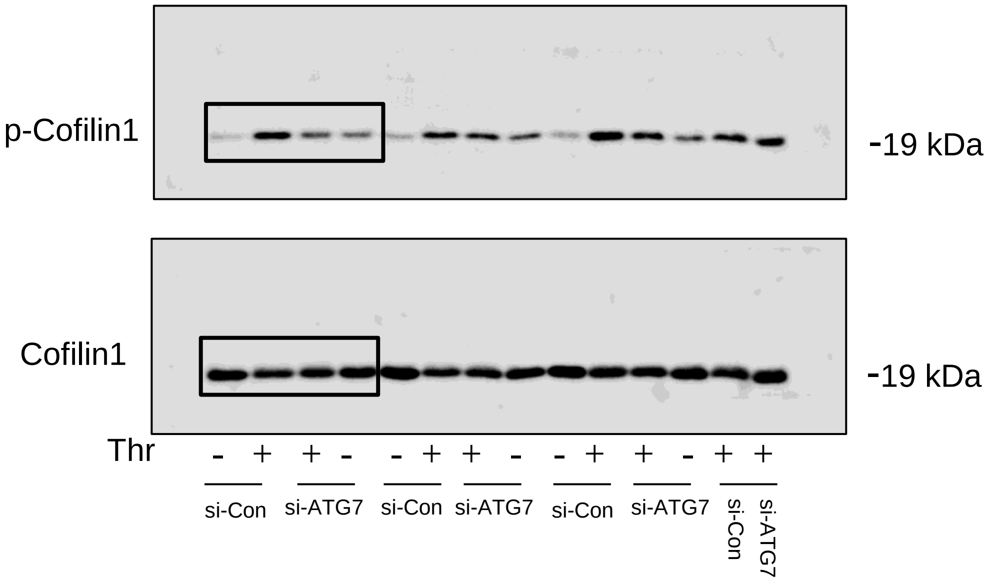

Figure S5. Uncropped versions of blots shown in Figure 4A. The area under the box was used in Fig. 4A.
